# Birth anthropometry and cord blood leptin in Korean appropriate-for-gestational-age infants born at ≥ 28 weeks’ gestation: a cross sectional study

**DOI:** 10.1186/s13633-020-00082-6

**Published:** 2020-06-26

**Authors:** Seok Jin Kang, Jin Gon Bae, Shin Kim, Jae Hyun Park

**Affiliations:** 1 Department of Pediatrics, Keimyung University Dongsan Medical Center, Keimyung University School of Medicine, Daegu, South Korea; 2 Department of Obstetrics and Gynecology, Keimyung University Dongsan Medical Center, Keimyung University School of Medicine, Daegu, South Korea; 3 Department of Immunology, Keimyung University School of Medicine, Daegu, South Korea

**Keywords:** Fetal growth, Leptin, Birth anthropometry

## Abstract

**Background:**

We investigated whether leptin during the third trimester was associated with fetal growth compared to IGF-1.

**Methods:**

One hundred five appropriate-for-gestational-age (AGA) infants born at ≥28 weeks’ gestation were enrolled. Cord blood leptin and insulin like growth factor 1 (IGF-1) were collected simultaneously during delivery. Enrolled infants were stratified into three groups according to GA as follows: 28 to < 34 weeks’ gestation, very preterm (VP); 34 to < 37 weeks’ gestation, late preterm (LP); and 37 to < 41 weeks’ gestation, term. Birth weight (BW), birth length (BL), head circumference (HC), and body mass index (BMI) were measured. Leptin and IGF-1 were logarithmically transformed to normalize their distributions in multivariable regression analysis.

**Results:**

Sixty-eight infants out of 105 infants were preterm (32.5 ± 2.5 weeks), and 37 infants were term (37.8 ± 1.2 weeks). BW, BL, HC, and BMI were higher with increasing gestational age among the three gestational age-specific groups. With regard to hormones, leptin and IGF-1 were higher with increasing gestational age. Log cord serum leptin was independently associated with BW and BL in multivariable linear regression analysis, after adjustment for confounding factors including gestational age, delivery mode, multiple pregnancy, pregnancy induced hypertension, gestational diabetes mellitus, infant’s BMI, and log cord blood IGF-1 levels.

**Conclusions:**

During the third trimester, cord serum leptin was independently associated with fetal growth.

## Background

Fetal growth results from complex interactions between fetal, maternal, and placental factors. However, the hormonal mechanisms of fetal growth are not completely understood. According to a previous study, the main endocrine regulator of fetal growth is the insulin-like growth factor (IGF) system [1]. Although growth hormone is the most important mediator of childhood growth, growth hormone plays a limited role in fetal growth because of the immature growth hormone receptor [2]. Fetal growth in early gestation is primarily regulated by IGF-2 expression, whereas IGF-1 is the dominant growth regulator in late gestation [2]. Thyroid hormones also stimulate intrauterine growth during the second half of gestation [3]. In the third trimester, fetal IGF-1 level increases steadily in coordination with the peak time of fetal weight gain [4].

Leptin is produced by adipose tissue and heavily involved in the control of energy balance, body weight, metabolism, and endocrine responses to fasting [5]. During gestation, leptin is mainly produced by three separate tissues (maternal adipose tissue, fetal adipose tissue, and placenta) [6]. As it has a high molecular weight (16 kDa), leptin produced by the placenta cannot cross the placental barrier and is not released into the maternal circulation [7]. This suggests that maternal and fetal leptin levels may be independent. Increasing evidence suggests that leptin has multiple roles in human physiology [7]. It is associated with osteoblast differentiation, whole-body bone mineral contents, hematopoiesis, and proliferation of yolk sac and fetal liver cells [8–10]. With regard to fetal growth, leptin in cord blood correlates with birth weight (BW) in a manner that seems to be independent of IGF-1 [11] (Christou et al., 2001), as well as with body fat mass, birth length (BL), head circumference (HC), ponderal index, and adiposity [12]. However, whether leptin is itself involved in fetal growth or just reflects fetal adiposity is unknown [13–15]. Furthermore, the major studies that reported an association between leptin and birth anthropometry were performed in term infants [11–15]. During the third trimester, which begins at 28 weeks of gestation, fetal weight increases more than fourfold [16]. Therefore, we investigated whether leptin during the third trimester was associated with fetal growth compared to IGF-1.

## Methods

This prospective study was conducted at Keimyung University Dongsan Medical Center, Daegu, South Korea, from October 2014 to May 2015. During the study period, mothers planning to give birth in our hospital were consecutively approached. Before delivery, we acquired informed consent from each pregnant patient for the collection of cord blood during delivery. Being a single-race study, both parents of enrolled infants were Korean. One hundred eighty-eight cases were recruited. Gestational age (GA) was estimated on the basis of the mother’s last menstrual period and ultrasonography findings. Because of the hormonal study of fetal growth in the third trimester, 23 cases of GA less than 27 weeks or more than 41 weeks were excluded. Considering sudden alterations in fetal hormones, we excluded 2 cases of suspicious perinatal asphyxia, with a 5-min Apgar score of < 6. Three cases with congenital anomaly were also excluded. We excluded seven cases in which no hormone was measured. Of the remaining 153 patients, one infant was dropped out of the study due to insufficient medical record. According to the standard Korean reference, AGA infants were defined as those whose birth weight for GA was between the 10th and 90th percentiles [17]. Forty-one infants with small-for-gestational age and six with large-for-gestational age were excluded. Finally, 105 appropriate-for-gestational age (AGA) infants born at 28–40 weeks’ gestation were enrolled. Enrolled infants were stratified into three groups according to GA as follows: 28 to ≤33 weeks’ gestation, very preterm (VP); 34 to ≤36 weeks’ gestation, late preterm (LP); and 37 to ≤40 weeks’ gestation, term. Anthropometric data (BW, BL, and HC) were measured within 2 h after birth by the nursing staff. Birth weight was recorded within 10 g using portable electronic scale. Birth length was obtained using a portable stadiometer accurate to 1 mm. Head circumference was measured between the glabella and the occipital prominence by a polyester non-extendable tape. To measure fetal adiposity, body mass index was calculated using weight (kg)/height^2^ (m^2^). Data on prenatal characteristics, including maternal pre-pregnancy body mass index (BMI), maternal pregnancy BMI, maternal age, 1- and 5-min Apgar scores, gestational diabetes mellitus, oligohydramnios, and pregnancyinduced hypertension were obtained from medical records.

The vein of a double-clamped segment of the umbilical cord was used to collect cord blood into a Vacutainer rapid serum tube (BD Bioscience, San Jose, CA, USA). Cord blood serum was separated and stored at − 20 °C until processing. Cord serum levels of leptin and IGF-1 were measured using magnetic bead-based multiplex immunoassays (Luminex 200, Merck Millipore Corp., Darmstadt, Germany). The study used Human Metabolic Hormone Magnetic Bead Panel multiplex panels (Cat. #HMHEMAG-34 K-03; Merck Millipore Corp., Darmstadt, Germany). For leptin, the limit of quantification of assays was 0.064 ng/mL and intra-assay coefficients of variation were < 10%. For IGF-1, the limit of quantification of assays was 19 pg/mL and intra-assay coefficients of variation were < 10%. The assays were completed in accordance with the manufacturer’s instructions.

Statistical analysis was done using SPSS version 20.0 (IBM Co., Armonk, NY, USA). Student t-test was used for comparison of anthropometric data and hormone levels which were expressed as mean ± standard error between two subject groups, one-way analysis of variance (ANOVA) was used to compare three subjects group. Post-hoc analysis was performed with Bonferroni method. Chi-square test was used for categorical variables. The relationship between birth anthropometry and cord blood hormones was evaluated using multivariate linear regression analysis. Because leptin and IGF-1 showed non-normal distribution, the variables were logarithmically transformed to normalize their distributions in multivariable linear regression analysis. A *p* value of < 0.05 was considered statistically significant.

## Results

Of the 105 enrolled infants in the study, 57 (54%) were male. Mean gestational age was 34.4 ± 3.3 weeks. Mean BW, mean BL, and mean HC, and mean BMI were 2399 g, 46.1 cm, 31.9 cm, and 10.99 kg/m^2^, respectively.

Table 1 compares prenatal characteristics, birth anthropometry, and cord blood hormonal levels between three GA-specific groups. Maternal pre-pregnancy BMI, maternal pregnancy BMI, maternal age, and the incidence of pregnancy induced hypertension and gestational diabetes mellitus were not different. With regard to birth anthropometry BW, BL, HC, and BMI were significantly different between three GA-specific groups (*p* < 0.05). With regard to hormones, there were significant differences in cord serum leptin and IGF-1 (*p* < 0.05). Cord serum leptin and IGF-1 in term infants were significantly higher than those in VP infants and LP infants (*p* < 0.05). Those of LP infants were also higher than those of VP infants (*p* < 0.05), as shown by ANOVA with post-hoc test.

**Table 1:** Anthropometric data and hormones according to gestational ages

	28–33 Weeks (*N* = 38)	34–36 Weeks (*N* = 31)	37–40 Weeks (*N* = 36)	*P-value*
Pre-pregnancy BMI (kg/m^2^)	22.8 ± 0.6	22.4 ± 0.8	21.9 ± 0.6	0.635
Pregnancy BMI (kg/m^2^)	27.0 ± 0.6	27.5 ± 0.8	27.7 ± 0.7	0.923
Maternal age (years)	34.5 ± 0.9	32.9 ± 0.7	33.9 ± 0.7	0.368
Multiple gestation	17 (48.6%)	11 (36.7%)	5 (14.7%)	0.009
Gestational diabetes, n (%)	7 (18.4%)	2 (6.5%)	1 (2.8%)	0.090
Oligohydroamnios, n (%)	0 (0%)	0 (0%)	1 (2.8%)	0.646
Pregnancy-induced hypertension, n (%)	3 (7.9%)	4 (9.7%)	1 (2.8%)	0.619
Birth weight (g)	1605 ± 63	2416 ± 51	3137 ± 37	< 0.001
Birth weight SDS	0.12 ± 0.1	− 0.28 ± 0.11	0.22 ± 0.1	0.003
Birth length (cm)	41.3 ± 0.5	47.2 ± 0.4	49.7 ± 0.3	0.009
Head Circumference (cm)	29.4 ± 0.5	32.2 ± 0.2	34.2 ± 0.2	< 0.001
BMI	9.36 ± 0.2	10.95 ± 0.17	12.7 ± 0.15	< 0.001
BMI SDS	0.23 ± 0.14	−0.31 ± 0.13	−0.04 ± 0.1	0.013
IGF-1 (ng/mL)	10.01 ± 0.68	14.80 ± 1.34	17.58 ± 1.18	< 0.001
Leptin (ng/mL)	2.13 ± 0.59	5.25 ± 1.31	7.68 ± 0.92	< 0.001

Continuous variables were expressed as mean ± standard error

Abbreviations. *BMI* Body mass index, *IGF-1* Insulin-like growth factor 1

We next compared prenatal characteristics, anthropometric data, and cord blood hormonal concentrations between males and females (Table 2). There was no significant difference in prenatal characteristics. BW, HC, and BMI were not significantly different between males and females. BL was significantly longer in males than in females (46.9 ± 0.5 vs. 45.1 ± 0.6 cm, *p* = 0.022). However, leptin and IGF-1 were not different between males and females.

**Table 2:** Prenatal characteristics, anthropometric data and cord blood hormonal concentrations between males and females

	Male (*N* = 57)	Female (*N* = 48)	*P-value*
Birth weight (g)	2435 ± 91	2308 ± 112	0.155
Birth weight SDS	0.17 ± 0.09	0.05 ± 0.09	0.766
Birth length (cm)	46.9 ± 0.5	45.1 ± 0.6	0.041
Head Circumference (cm)	32.3 ± 0.3	31.6 ± 0.4	0.199
BMI	10.9 ± 0.2	10.9 ± 0.3	0.661
BMI SDS	−0.2 ± 0.09	0.1 ± 0.13	0.040
IGF-1 (ng/mL)	14.26 ± 0.92	13.74 ± 1.04	0.712
Leptin (ng/mL)	4.69 ± 0.81	5.23 ± 0.85	0.628

Abbreviations. *BMI* body mass index, *IGF-1* insulin-like growth factor 1

A linear regression analysis between birth anthropometry and cord serum hormones was presented in Table 3. Cord serum leptin and IGF-1 were logarithmically transformed to normalize their respective distributions. Log cord serum leptin and IGF-1 positively correlated with BW, BL, HC, and BMI in the univariable analysis (*p* < 0.05). Log cord serum leptin were independently associated BW, BL, and HC in the multivariable regression analysis, after adjustment for gestational age, delivery mode, multiple gestation, pregnancy induced hypertension, gestational diabetes mellitus, infant’s BMI, and log cord serum IGF-1. Similarly, log cord serum IGF-1 were independently associated with BW and BL in the multivariable regression analysis, after adjusting for gestational age, delivery mode, multiple gestation, pregnancy induced hypertension, gestational diabetes, infant’ BMI, and log cord serum leptin.

**Table 3:** Results of linear regression analysis for determinants of birth anthropometry in infants

Variables		Univariate analysis			Multivariate analysis	
		R^2^	*p-value*	adjusted R^2^	β	*p-value*
BW (g)	GA (weeks)	0.88	< 0.001			
	Log IGF-1 (ng/mL)	0.31	< 0.001	0.90	334.94	0.020
	Log Leptin (ng/mL)	0.52	< 0.001	0.90	194.888	0.006
BL (cm)	GA (weeks)	0.80	< 0.001			
	Log IGF-1 (ng/mL)	0.24	< 0.001	0.69	3.579	0.018
	Log Leptin (ng/mL)	0.46	< 0.001	0.69	2.344	0.002
HC (cm)	GA (weeks)	0.68	< 0.001			
	Log IGF-1 (ng/mL)	0.15	< 0.001	0.61	−0.05	0.962
	Log Leptin (ng/mL)	0.40	< 0.001	0.61	0.88	0.092
BMI (g/cm^3^*100)	GA (weeks)	0.005	0.480			
	Log IGF-1 (ng/mL)	0.015	0.214	0.08	0.39	0.358
	Log Leptin (ng/mL)	0.001	0.712	0.08	0.18	0.414

Abbreviations. *BW* body weight, *GA* gestational age, *BL* body length, *GA* gestational age, *HC* head circumference, *IGF-1* insulin-like growth factor 1

## Discussion

In the present study, cord blood leptin and IGF-1 were independently associated with BW and BL. Regarding BW, Christou et al. [11] reported that leptin and IGF-1 in cord blood significantly correlated with BW after adjustment for sex, gestational diabetes mellitus, pregnancy induced hypertension, and maternal smoking. Vatten et al. [12] reported that cord blood leptin and IGF-1 in term infants contributed independently and positively to BL and BW. Varvarigou et al. [14] reported the association between BW and cord blood leptin in AGA term infants. Yang et al. [15] reported that BL in term infants significantly correlated with cord blood IGF-1 and leptin, but not with cord blood insulin level. Compared to previous studies [11–15], the strength of our study is that we enrolled preterm infants as well as term infants to investigate the relationship between cord serum leptin and earlier birth anthropometry. Because fetal weight increases rapidly during the third trimester [16], we enrolled infants born at ≥28 weeks’ gestation. In the present study, the prevalence of preterm infants was 64.8% and mean gestational age was 32.5 weeks. Compared to previous studies that reported correlation between cord blood leptin and birth anthropometry in preterm infants [18, 19], we included a larger number of preterm infants and earlier gestational age preterm infants. The second strength of the present study is that only AGA infants with single ethnicity were enrolled to exclude potential confounders in SGA infant. Third strength is we enrolled single race and ethnicity because birth weight varies by race and ethnicity [20–22].

Known placental hormones affecting the fetal growth include estrogens, progesterone, human chorionic gonadotropin. The role of leptin as a regulator of fetal growth has not been elucidated. Leptin levels may simply reflect adipose tissue mass because cord blood leptin at birth was associated with fat mass index positively [23]. Infants of appropriate birth weight who have congenital leptin gene mutation or leptin receptor gene mutation may suggest that birth anthropometry is not related to leptin [24, 25]. However, leptin receptors expressed in fetal rat pancreatic islet cells [26] and in human fetal gastric mucosa may suggest that leptin is involved in organ growth [27]. Fetal leptin increases bone mass [28]. Leptin administration promoted lung maturation in the ovine fetus [29]. In the present study, we utilized BMI to adjust for adipose tissue mass. After adjustment for BMI in multivariate regression analysis, the odds ratio of cord blood leptin levels for BW and BL remained significant (*p* < 0.05). Given these considerations, leptin may not simply reflect mass of adipose tissue but participate directly in fetal growth. However, BMI is not a direct method for measuring body composition. Methods of body composition assessment in the infant include dual energy X-ray absorptiometry, air displacement plethysmography, and bioelectrical impedance analysis. Although BMI SDS has been shown to be a good predictor of fat mass [30] and useful anthropometric index for body fat percentage in infants [31], care should be taken in interpreting the results.

Female fetuses grow slower than male fetuses during the second and third trimesters [32]. Female infants have higher cord blood leptin levels than male infants [15, 32–34]. Contrary to previous reports, no significant differences in birth anthropometry and cord blood leptin level were found between males and females [19, 35, 36]. In the present study, a significant sex-specific difference was noted in BL, but cord blood leptin and IGF-1 were not different between males and females.

The limitations of the present study were that we did not investigate other hormonal confounders which may affect fetal growth or umbilical leptin levels including IGF-2, IGFBP-1, glucocorticoid and insulin. Early fetal growth is primarily regulated by IGF-2, and cord blood IGFBP-1 has been associated with low BW [12]. Antenatal administration of exogenous glucocorticoid or dexamethasone might increase umbilical leptin which is intervention to reduce the severity of neonatal respiratory morbidity [36]. Considering the inclusion of preterm infants in the present study, not fully evaluated history of antenatal steroid administration can be a confounder of the present study. Insulin may regulate leptin levels through both adipoinsular axis and IGF-1 [37]. Previous reports suggested that insulin stimulates leptin secretion in vitro and in vivo [14]. However, Fonseca et al. [19] reported that birth anthropometry in preterm infants showed a significant association with cord blood leptin, but not with cord blood insulin. Additional studies with large number of infants are needed to clarify associations between cord blood leptin levels and birth anthropometry. Long-term data are also needed to investigate effect of leptin on postnatal growth.

## Conclusion

During the third trimester, cord serum leptin was independently associated with birth anthropometry.

## Data Availability

The datasets during and/or analyzed during the current study available from the corresponding author on reasonable request.

## Abbreviations

AGA: Appropriate-for-gestational age
BMI: Body mass index
BW: Birth weight
BL: Birth length
GA: Gestational age
HC: Head circumference
IGF: Insulin-like growth factor
LP: Late preterm
PROM: Premature rupture of membrane
SGA: Small- for-gestational-age
VP: Very preterm
